# Not just a gut feeling: a deep exploration of functional bacterial metabolites that can modulate host health

**DOI:** 10.1080/19490976.2022.2125734

**Published:** 2022-09-20

**Authors:** Andrew Gold, Jiangjiang Zhu

**Affiliations:** Human Nutrition Program & James Comprehensive Cancer Center, The Ohio State University, Columbus, OH, USA

**Keywords:** Microbial metabolites, host health, mechanism, metabolomics

## Abstract

Bacteria have been known to reside in the human gut for roughly two centuries, but their modulatory effects on host health status are still not fully characterized. The gut microbiota is known to interact with dietary components and nutrients, producing functional metabolites that may alter host metabolic processes. The majority of thoroughly researched and understood gut microbial metabolites fall into two categories: short-chain fatty acids (SCFAs) and bacterial derivatives of dietary tryptophan. Despite the heavy emphasis on these metabolites, other metabolites stemming from microbial origin have significant impacts on host health and disease states. In this narrative review, we summarize eight recent studies elucidating novel bacterial metabolites, detailing the process by which these metabolites are identified, their actions within specific categories of human health, and how diet may impact production of these metabolites. With similar future mechanistic research, a more complete picture of bacterial impact on host metabolism may be constructed.

## Introduction

**Figure 1.**
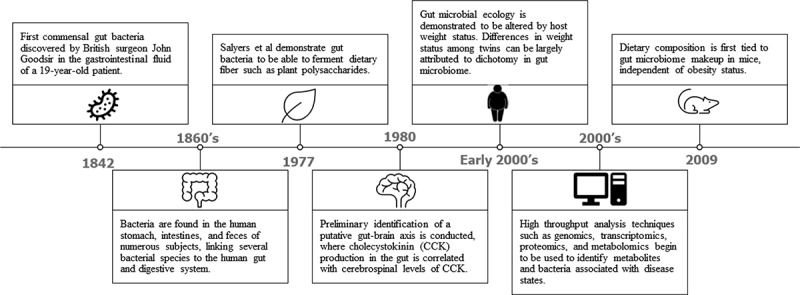
A brief history of the discovery/ analysis of gut microbes and the implications of their metabolites to host function.^1–7^

The human gut microbial population has long been known to influence host metabolism, from its assistance in drug metabolism to microbially induced alterations in weight status and cancer pathogenesis (Figure 1). Early experiments identifying the existence of bacteria in the gut were first conducted in the mid-19^th^ century, but discovery of the exact mechanisms by which human intestinal microflora alter host metabolic processes has been a gradual process.^1^ Throughout the 19^th^ and 20^th^ centuries, human gut flora were increasingly associated with host metabolism. For instance, gut bacteria were shown to degrade dietary fiber in 1977, when it was demonstrated that several species of gut-derived *Bacteroides* were able to ferment plant polysaccharides and mucin.^2^ In the early 20^th^ century, it was discovered that the genetic makeup of the gut microflora could be correlated with host weight status, indicating that variations in bacterial composition in the gut could regulate host phenotypes.^3,4^ Soon thereafter, dietary composition was correlated with bacterial species abundance in the gut, demonstrating that host metabolism may conversely affect the microbial makeup of the gut.^5^ Additionally, many disease states, such as neurological disorders, obesity, cardiovascular disease, and even allergies have been shown to be associated with “dysbiosis”, the perturbation of the gut microbial composition from that of healthy subjects.^8^ It was soon discovered that bacteria could metabolize dietary substances into their own metabolites, which may modulate host metabolic processes by acting as pseudo-synthetic ligands for host enzymes and receptors.^9–11^ Studies focusing on bacterial metabolite production have largely focused specifically on the production and activities of bacterially produced short-chain fatty acids (SCFAs), which result from the fermentation of dietary fiber, as well as bacterial metabolism of circulating tryptophan, which can produce products impacting far-reaching fields such as cancer metabolism, cardiovascular health, and neurological function.^12,13^ While these metabolites are no doubt important in the grand scheme of human health, they represent a small fraction of those synthesized by intestinal microbiota, the majority of which have been underexplored. Few studies, then, have conducted detailed identification and mechanistic validation of singular bacterial metabolites aside from SCFAs and mainstream tryptophan metabolites in specific facets of host metabolism.

**Figure 2.**
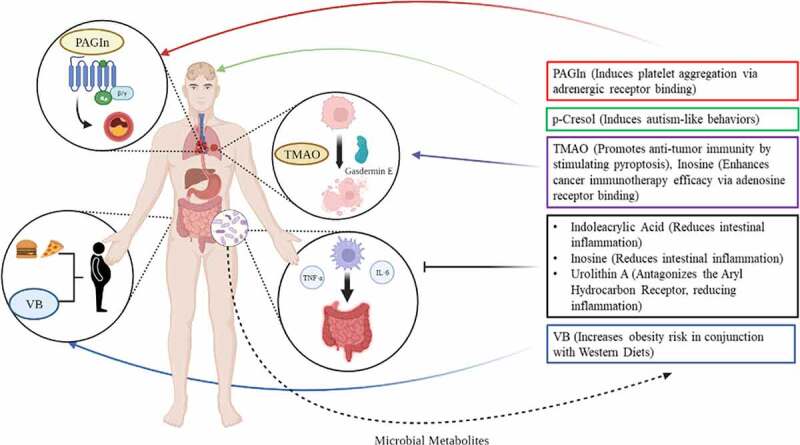
Identified metabolites stem from gut bacterial production but have far reaching impacts across multiple tissues and organ systems in the host. TMAO = Trimethylamine N-oxide, PAGln = phenylacetylglutamine, VB = delta valerobetaine. Figure generated using BioRender software (BioRender.com).

Studies that have conducted detailed mechanistic investigations, however, have made monumental advances in understanding the processes whereby gut microbes may alter host metabolism. In this narrative review, we describe several such studies that were able to discover novel bacterial metabolites that have modulatory effects on host metabolic health and disease states (Table 1, Figure 2). While these metabolites are known to exist, their clear microbial origins and downstream effects to the host detailed by these studies present innovative understanding of how particular bacterial species and metabolites may impact human health via defined mechanisms. In this review, we detail similarities in methodological approaches, contextualize the discovery of novel metabolites in our identified studies, and discuss these exciting new developments in paving clear pictures of microbial metabolite-driven host responses.

**Table 1.** Microbial metabolites and their target of action identified across eight mechanistic studies.

Metabolite	Metabolite Target	Major Impact	Effect on Human Health	Metabolite Class		Reference Number
**Delta-valerobetaine**	Mitochondrial B-oxidation	Inhibits mitochondrial carnitine shuttle and alters PPARα signaling	Increases Adiposity	Straight Chain Fatty Acid		15
**Phenylacetylglutamine**	B-adrenergic receptors	Induces CVD risk factors including platelet adhesion and aggregation via adrenergic receptor binding	Leads to elevated risk of CVD	Amino Acid		17
**Indoleacrylic Acid**	Immune Cells, Goblet Cells	Reduces inflammatory markers while promoting goblet cell differentiation	Reduced inflammation in gut	Monocarboxylic acid, Indole derivative		16
**Inosine**	T-cells	Assists in anti-tumor immunity by modulating T-cells in an A2AR-dependent manner	Improved efficacy of cancer immunotherapy	Purine Nucleotide		19
**Inosine**	Intestinal Goblet Cells	Promotes gut health and decreases mucosal inflammation in ulcerative colitis in an A2AR/PPARγ dependent manner	Improved ulcerative colitis phenotypes	Purine Nucleotide		20
**Urolithin A**	Aryl Hydrocarbon Receptor	Acts as an AhR antagonist	Decreased inflammation and numerous other cellular functions		Coumarin	27
**Trimethylamine N-oxide**	T-cells	Increases efficacy of tumor immunotherapy in triple-negative breast cancer	Improved efficacy of cancer immunotherapy in triple-negative breast cancer	Tertiary Amine Oxide		18
**p-Cresol**	Dopaminergic Neurons	Alters social behavior toward autism-like patterns when administered to mice	Altered social behaviors	Cresol		14

Foodnotes: PPARα = Peroxisome Proliferator Receptor Alpha, PPARγ = Peroxisome Proliferator Receptor Gamma, A2AR = A2A Adenosine Receptor

## A common workflow for identification of microbially derived metabolites

**Figure 3.**
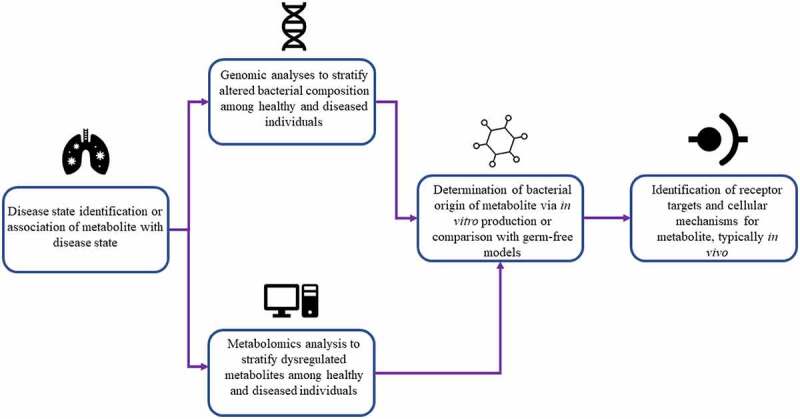
A generalized workflow for metabolite detection based on commonalities identified in analytical techniques employed in the studies we have identified.

In the studies we identified, we were able to consolidate a general overarching workflow for analytical determination of specific bacterial metabolites implicated in human health and disease states. A general schematic for this workflow is depicted in Figure 3.

The primary method of metabolite identification across the analyzed studies is to place emphasis on a particular disease state known to be modulated by microbes or associated with a specific microbial metabolite. Selection of a focal metabolic state allows for targeted stratification of dysregulation and comparison between healthy controls in the second major stage of metabolite identification. Then, high throughput (omics) analyses were conducted to discriminate dysregulated metabolites or bacterial species. For example, untargeted metabolomics analyses were conducted to some degree, either by the authors or extracted from previous analyses, in several reported studies (Table 1).^14–20^ Metabolomics, defined as “the comprehensive analysis of metabolites (small molecule intermediates or end products of metabolic processes) in a biological specimen”, is an emerging technique that allows for up to the minute phenotypic profiling of an organism’s metabolic state.^21^ Metabolomics can be a powerful tool for identification of metabolites or metabolic panels that can be used as biomarkers for various disease states, including cancers, neurodegenerative diseases, and metabolic abnormalities.^22–24^

The other major method of high throughput analysis conducted by our identified studies is that of bacterial genotyping via 16S rRNA or metagenomic sequencing, utilized to identify and compare presence of certain bacterial species across experimental conditions. These methods typically compare aberrations in mostly conserved genes (usually the gene coding for 16S ribosomal RNA) that can be associated with particular bacterial taxa in order to identify species abundance in an isolated sample.^25^ While fewer studies employed this method as a primary identification method, genomic sequencing has advantages in its ability to identify particular taxonomic groups that can produce a given metabolite, as many metabolites can be synthesized only by a subset of bacterial species containing a particular biosynthetic gene cluster.^26^ While only Wang et al. and Li et al. conducted 16S sequencing as their primary stratification method, Mager et al. and Nemet et al. conducted genomic analyses following metabolomics to associate production of their identified metabolites with specific bacterial species.^17–20^ In addition to identification of bacterial species that may produce a given metabolite, whole genome shotgun sequencing may have more specific applicability in certain studies. For example, Wang et al., focusing on microbial modulation of immunotherapy treatment in triple-negative breast cancer, employed 16S sequencing to demonstrate the presence and taxonomic composition of bacterial species that were able to colonize tumor tissue, which led to the identification of a metabolite implicated in cancer immunotherapy.^18^

Lastly, in studies describing microbial production of metabolites that affect host metabolism, it is integral to prove these metabolites are indeed of bacterial origin and produced in physiologically relevant concentrations by the microbiota. Metabolites were either identified to be produced by bacteria through various methods or, in the case of urolithin A and p-Cresol, were compounds known to stem largely from bacterial production.^14,27^ Metabolites that were identified to be produced by microbiota during the course of the study were either identified via comparison of conventional animals to germ-free or pseudo germ free animals created by antibiotic administration,^15,17,19,20^ or from *in vitro* incubation of specific bacterial strains and subsequent mass spectrometric identification of metabolites in media supernatant.^16,18^ With these discussed methods, metabolites specifically produced by gut bacteria can be identified and correlated with human health or disease states in a systematic fashion.

## Receptor targets of gut bacterial metabolites and downstream effects of the host

Bacterial metabolites with profound effects are largely mediated by major receptors implicated in target diseases. Here, we will discuss several major signaling or metabolic pathways impacted by bacterial metabolites and receptors that mediate these effects.

### Novel metabolites and receptors implicated in colonic inflammatory processes

Gut microbes have long been known to mediate inflammation in the gut. SCFAs, and kynurenine, a common product of bacterial tryptophan metabolism, have consistently been tied to the chemoattraction of inflammatory mediators such as macrophages, lymphocytes, and plasma cells to the intestinal epithelia.^28^ Additionally, some gut bacteria phyla such as Proteobacteria stimulate inflammatory response simply by virtue of their Gram-negative specific lipopolysaccharide (LPS) as a major component of their cell wall, which is recognized by macrophages and spurs generation of inflammatory factors such as tumor necrosis factor alpha (TNF-α), IL-6, and IL-1.^29^ Other phyla of gut bacteria, such as Firmicutes, conversely, can contribute to anti-inflammatory processes via production of metabolites or proteins that can lower the inflammatory response.^30,31^ In the studies that were identified for this review, microbially produced urolithin A, indoleacrylic acid, and inosine were associated with reduced inflammation, largely within the gut mucosa (Figure 2).^16,20,27^ The effects of these three metabolites were mediated by two distinct receptor systems.

In the study conducted by Muku et al., urolithin A was determined via dose-dependent experiments to act as a direct aryl hydrocarbon receptor (AhR) antagonist.^27^ AhR is a transcription factor implicated in a wide array of diseases, including cancer pathogenesis, neurologic inflammation, and, most importantly for the gut, activation of macrophage produced inflammatory factors such as IL-10, IL-22, and prostaglandin E2, and potential exacerbation of ulcerative colitis and Crohn’s disease symptomology, often in response to microbially secreted factors.^32–34^AhR can conversely also act to suppress inflammation and has been shown to do so in such diseases as nonalcoholic fatty liver disease (NAFLD).^35^ Its mediation of inflammation is largely ligand and pathway-dependent. For example, in the gut, endogenous ligands such as kynurenic acid and indoxyl sulfate activating inflammatory pathways, while other ligands such as lipoxin A4 may activate AhR to resolve inflammation.^35–37^ Additionally, dietary components such as quercetin and galangin may act either directly or indirectly to activate AhR.^35^ AhR has not been fully characterized, and many new ligands, both endogenous and microbially synthesized, are still being discovered.^35^ Urolithin A, the vast majority of which is synthesized by the microbiota, has additionally been recently shown to attenuate neuroinflammation in a mouse model of Alzheimer’s Disease.^38^ While this study did not directly link this decrease in inflammation to AhR, it is noteworthy that AhR has been shown to be upregulated in postmortem analyses of hippocampal tissue in Alzheimer’s patients.^39^

In addition to urolithin A, indoleacrylic acid has been shown to mediate anti-inflammatory processes through AhR mediation. Wlodarska et al. found commensal *Peptostreptococcus* can metabolize tryptophan to indoleacrylic acid, which is able to stimulate Nuclear factor-erythroid factor 2-related factor 2 (NRF2), a transcription factor regulating a host of downstream factors including those responsible for antioxidation and detoxification.^16,40^ NRF2 upregulates, among other downstream genes, AhR. Wlodarska et al. showed that indoleacrylic acid resulted in AhR-mediated upregulation of mucin-related genes such as Muc2, resulting in decreased inflammation and increased goblet cell proliferation, integral for protection against mucosal inflammatory diseases such as ulcerative colitis.^16^

Lastly, the microbial metabolite inosine (a purine nucleotide), likely produced by *Lactobacillus* species in the gut from fermentation of barley leaf, was demonstrated by Li et al. to mediate inflammation through the A_2A_R (adenosine receptor)/PPARγ (peroxisomal proliferator-activated receptor gamma) pathway.^20^ While A_2A_R’s primary ligand is adenosine, it exhibits promiscuity toward other nucleotides, including the deaminated product of adenosine, inosine.^39^ A_2A_R activation typically results in anti-inflammatory properties via cAMP response element binding protein (CREB) inactivating inflammatory marker NF-κβ.^41^ It may also activate the downstream transcription factor PPARγ via protein kinase A. PPARγ proliferates its anti-inflammatory effect by upregulating lipid synthesis genes leading to anti-inflammatory eicosanoid production.^42^ Thus, microbially produced inosine, which was shown to be significantly increased in production with the feeding of barley leaf, can activate A_2A_R and result in reduced inflammation.^20^ Additionally, inosine was found to increase Muc2 expression in a PPARγ-dependent fashion.^20^ Thus, altogether, microbial metabolites produced by commensal species may contribute to reduction of inflammation in the gut, leading to potential improvement of inflammatory diseases such as ulcerative colitis and Crohn’s disease.

### Gut microbial metabolites improve efficacy of cancer immunotherapy

In addition to modulation of colonic inflammation, metabolites produced by gut microbes can be immunomodulatory, specifically in the context of cancer immunotherapy. Cancer immunotherapy is an emerging therapeutic option that better equips the cells of the adaptive immune system to recognize and eliminate cancer cells. While immunotherapy represents a promising alternative to chemotherapy, its effectiveness is heterogenous and may invoke toxicity in some patients.^43,44^ One theory for this variety in response is the variability in the gut microbial composition of individual subjects. This theory is furthered by the knowledge that fecal microbiota transplants (FMTs) have been shown to improve efficacy of immunotherapy as well as reduce toxicity effects, leading to hypotheses that the microbiota may be somehow modulating inter-individual responses to immunotherapy.^43^ In our identified studies, two metabolites were found to improve efficacy of immunotherapy.

First, inosine, previously shown to modulate inflammatory response in the gut by binding to A_2A_R receptors, was shown to improve response to checkpoint inhibitor immunotherapy through the same receptor system.^19^ Interestingly, this positive immunoregulatory effect would be considered pro-inflammatory, while A_2A_R binding typically leads to anti-inflammatory effects via protein kinase A, as demonstrated by Li et al.^20^ Mager et al. demonstrated that inosine produced by *Bifidobacterium pseudolongum* was able to exert a pro-inflammatory effect on improving efficacy of cancer immunotherapy in conjunction with interferon gamma (IFN-γ), where IFN- γ + T cells demonstrated increased splenic presence in mice and exogenous IFN- γ enhanced the anti-tumorigenic properties of inosine *in vitro*. Furthermore, inosine was shown to mediate these anti-tumor immune effects *only* via T cells, as direct administration of inosine to MC38 tumor cells did not alter tumor viability.^19^ In conjunction with anti-CLA-4 therapy, inosine and inosine-producing bacterial species were able to, via A_2A_R-mediated effects on T cells, reduce tumor size significantly more than anti-CLA-4 therapy alone. Altogether, it was found that microbially produced inosine acting on A_2A_R receptors, which typically induce anti-inflammatory responses, may elicit pro-inflammatory effects that increase the efficacy of cancer immunotherapy treatment.

In addition to inosine, microbial metabolite trimethylamine N-oxide (TMAO), typically cited as a risk factor for cardiovascular health and found to be correlated with cardiovascular mortality,^45^ may also modulate efficacy of cancer immunotherapy. Wang et al. first demonstrated, using 16S sequencing, that bacteria, including those belonging to the genera *Bacteroides, Psuedomonas, Acinetobacter, and Dermacoccus*, may colonize tumor tissue in triple-negative breast cancer.^18^ They next demonstrated that tumor-resident microbes, when cultured, synthesized high amounts of TMAO. Further experiments demonstrated TMAO increases anti-tumor immunity by enhancing the function of CD8 + T cells, largely through induction of pyroptosis by upregulating pyroptotic markers Gasdermin E (GSDME) and downstream kinase protein kinase R-like endoplasmic reticulum kinase (PERK), which functions as an endoplasmic reticulum stress sensor (Figure 2).^18,46^ This study is important for two reasons. First, it demonstrates tumor-resident microbes may secrete factors modulating therapeutic response. Second, it demonstrates microbially produced TMAO can lead to improved health outcomes in non-CVD related cases. Altogether, these two studies show that microbially produced metabolites such as inosine and TMAO may be used to mechanistically explain how the gut microbiota modulate the well-documented inter-individual variation in response to cancer immunotherapy.

### Bacterial metabolites implicated in obesity and cardiovascular disease

In addition to modulating immune status and cytokine production, the gut microbiota may influence more general metabolic functions such as host weight and cardiovascular status through their production of metabolites. Gut microbes can alter weight status through a variety of mechanisms. First, weight status has been shown to have bearing on bacterial composition in the gut. While certain bacterial phyla and species have been correlated with obese subjects, these studies have not proven whether this taxonomic association comes as a driver or a consequence of obesity.^47^ Conversely, FMT of bacteria derived from obese mice to wild-type mice causes the wild-type to extract more calories from their food resulting in a greater increase in body fat than in mice given FMT from lean mice.^48^ Hypotheses for this modulation of weight status largely revolve around the effects of SCFAs or microbially produced ethanol, all of which can impact mitochondrial function and alter energy production processes.^49^ While obesity has significant impact on cardiovascular disease, resident bacteria have additionally been shown to modulate cardiovascular state independent of weight status. Gut microbiota may regulate cardiovascular disease (CVD) via multiple mechanisms, including production of secondary bile acids which may exert differential effects on blood pressure and arterial function; SCFA production, which may facilitate blood pressure alterations dependent upon expression of g-protein coupled receptors (GPCRs) such as Gpr41 and Olfr78; and TMAO, which has been significantly implicated in cardiovascular mortality and atherosclerosis.^45,50^ Thus, while previous literature has identified the gut microbiota to be significantly associated with both weight status and cardiovascular health, only a few microbial metabolites have thus far been associated with either category. Here, we describe two novel functional microbial metabolites, one linked to obesity and another linked to cardiovascular disease outcome.

First, Liu et al. discovered a microbially manufactured metabolite, delta-valerobetaine (VB), was able to impair mitochondrial β-oxidation, leading to accumulation of circulating long chain fatty acyl CoA.^15^ This metabolite was shown to impair fatty acid oxidation in mice, while subsequently eliciting upregulation of downstream genes of peroxisomal proliferator-activated receptor alpha (PPARα), which is somewhat puzzling given the role of PPARα in promoting lipolysis.^15,51^ However, this trend was reversed with the feeding of a “Western Diet”, consisting of high fat and sugar chow, which, when administered concomitantly with VB, led to an increase in perigonadal visceral adipose tissue, posterior subcutaneous adipose tissue, and interscapular brown adipose tissue, as well as exacerbated hepatic steatosis.^15^ Similar trends were observed in human subjects in a clinical setting, where increased plasma VB was correlated with increased visceral adipose tissue, increased BMI, and increased incidence of hepatic steatosis.^15^ This finding suggests that not only are gut microbes able to alter host weight status through mechanisms unrelated to CVD, but these mechanisms are also significantly modulated by host diet.

Additionally, Nemet et al. identified a gut microbe-derived metabolite, phenylacetylglutamine (PAGln), to be significantly associated with adverse cardiac events in a large cohort. This metabolite was then shown to induce platelet aggregation and enhance submaximal ADP-stimulated P-selectin surface expression dose-dependently.^17^ Furthermore, it was demonstrated to dose-dependently enhance thrombin-stimulated increases in intracellular calcium concentrations.^17^ This effect was determined to be modulated by PAGln’s stimulation of adrenergic receptors, which lead to phospholipase C activation and modulation of calcium efflux through inositol phosphate (IP3)-gated channels, stimulating platelet aggregation and adhesion to collagen matrices (Figure 2). The above effects combined lead to platelet aggregation, thrombosis, and worsened CVD outcomes.^17^ PAGln represents the second identified metabolite, in addition to inosine, to act via adrenergic receptors, but the first to stimulate disease-related effects in the host. Notably, this study offers evidence that microbial metabolites aside from TMAO may significantly promote CVD. Altogether, these studies prove that microbes may modulate host core metabolic processes such as circulation and weight status through non-canonical metabolites.

### Bacterial metabolites that may alter brain or nervous system function

An emerging topic in the study of gut microbes focuses on their ability to affect brain and nervous system function (Figure 2), in what is termed by many researchers as the ‘gut-brain axis’. This field has largely been constructed from the understanding that bacterial tryptophan metabolism and relative synthesis of serotonin precursor 5-hydroxytryptophan (5-HT), when compared to production of indoles or kynurenine, can elicit demonstratable effects on brain and nervous system activity. Bacterially produced 5-HT can then regulate host serotonin availability and biosynthetic activity.^52^ Additionally, microbially produced SCFAs may regulate neurochemistry and microglial homeostasis via AhR binding.^53^ These bacterial products may all signal to the nervous system and play a significant role in the development and progression of neuropsychiatric disorders such as Alzheimer’s Disease, Schizophrenia, and Autism Spectrum Disorder (ASD).^53^ While this gut-brain axis has been a focal point for microbiome research in recent years, much is still not understood. For instance, exact mechanisms for the pathogenesis of Alzheimer’s Disease and ASD have not been completely elucidated, likely because these diseases actually stem from multiple pathologies with similar symptomology. Because this field is complicated and in its relative infancy, metabolites beyond SCFAs and tryptophan metabolites are even more unexplored than in other fields related to microbial metabolism.

In 2021, Bermudez-Martin et al. discovered p-Cresol, known as a potentially toxic uremic solute, to be present in heightened concentrations in the urine and feces of ASD patients. Interestingly, this association was not identified in any blood-related specimen.^14^ When p-Cresol was administered directly to mice, it elicited a similar effect, as the metabolite was identified in only stool and urine but not plasma. When administered, p-Cresol induced autism-like behaviors in mice, including reduced social behaviors and increased incidence of stereotypies such as head shaking and circling, while notably demonstrating absence of true cognitive impairment.^14^ p-Cresol urinary levels were additionally correlated with severity of ASD symptomology. Mechanistically, this study was not able to identify a particular receptor responsible for the effects of p-Cresol on the nervous system, in contrast with other identified metabolites. This may be due to the lack of clearly identified mechanisms for ASD development outside of abnormal neural connectivity. However, Bermudez-Martin et al. were able to demonstrate that p-Cresol impairs dopamine neuron excitability and connectivity in the brain’s ventral tegmental area, and that p-Cresol elicits significant alterations in gut microbial composition. Importantly, transplantation of normal microbiota to p-Cresol treated mice restores the normal function of most social behavior.^14^ Thus, this microbially produced metabolite has significant bearing on autism-like behavior, where it impacts the function of the ventral tegmental area of the brain, and this behavior modification is in some way dependent on the metabolite’s ability to alter microbiome composition.

## Many microbially produced metabolites are directly impacted by host dietary intake

As the gut microbiota reside in the intestines, they have proximal access to dietary components, particularly those that are not properly absorbed in early stages of the gastrointestinal tract. These dietary factors and nutrients have profound influence on the metabolites produced by the microbiota. For instance, a 2016 study comparing microbiome profiles of vegans and omnivores found significant metabolic variation between the two groups stemming from the microbiota but little taxonomic variation in bacterial species.^54^ Dietary fiber, essentially a catch-all term for indigestible carbohydrates of plant origin, is known to be fermented by certain bacterial species in the gut to produce metabolites such as SCFAs. Recent studies have demonstrated that not only does fiber quantity of the diet impact SCFA production, but various fiber types may be preferred by different species of bacteria in the gut, which, as different species have distinct fermentation patterns, would produce differing SCFA quantities and thus alternative modulations on host metabolism.^55^ The metabolites identified by our review may also differ in production quantities or activities dependent on host dietary patterns. For instance, barley leaf was directly identified to stimulate inosine production and relieve intestinal colitis by Li et al.^20^ Western-style diets were found by Liu et al. to alter the activity of microbially produced VB to shift metabolic profiles toward exacerbated obesity and impaired fatty acid oxidation.^15^ PAGln is produced by bacterial biotransformation of dietary phenylalanine and subsequent conjugation with glutamine, p-Cresol produced by bacterial tyrosine metabolism, and indoleacrylic acid produced by bacterial metabolism of dietary tryptophan, all of which suggest dietary proportions of certain amino acids can significantly alter the incidence and progression of disease states.^16,17,56,57^ Urolithin A is produced from bacterial multi-step processing of ellagic acid, which is a dietary polyphenol found in a variety of fruits including strawberries, cherries, and blackberries, as well as nuts such as walnuts and pecans.^27^ Lastly, TMAO, which has differing effects on CVD and efficacy of immunotherapy for triple-negative breast cancer, is generated from dietary choline, betaine, and L-carnitine, all of which are found mostly in animal products such as meats, eggs, and dairy products.^58^ Interestingly, many of these metabolites stem from either solely animal products or solely plant products. This has been shown to induce significant differentials in production of certain bacterial metabolites, such as p-Cresol, between vegetarians and omnivores.^57^ This suggests that not only consumption of specific foods such as barley leaf, but overarching dietary patterns may contribute to differential microbial metabolic processes and then eventually host health. While consumption of nutrients leading to production of any one of these compounds may significantly influence probabilities of disease state progression, each of these nutrients have multiple alternative effects unrelated to microbial metabolism. Additionally, inter-individual variation in gut microbiome composition may alter relative production of each of these metabolites even when the same diet is consumed.^59^ Lastly, it is important to consider that while certain metabolites may be produced in higher quantities by certain bacterial species or genera, these genera may not be wholly characterized by their production of this metabolite. For instance, while bacterial genera such as *Bifidobacteria* may produce metabolites such as inosine that can spur increased inflammation, other metabolites produced by this genera, such as aromatic lactic acids, have been found to demonstrate distinct anti-inflammatory properties.^60^ It is thus important to recognize the complexity of each bacterial species with regard to producing specific metabolites. Consequently, those with similar gut microbiomes may still be affected differently by the bacteria in their gut dependent upon diet quality. Therefore, precision nutrition with microbial metabolism in mind may be warranted in the future, especially when considering those already at risk for certain disease states.

## Research limitations and future directions

While each of the studies we have outlined represents a monumental advance in the understanding of how gut microbes may modulate human health by production of novel metabolites, conduction of studies such as these with detailed mechanistic explication represents an incredibly difficult and arduous task. Moreover, identification of metabolites with significant association to human health and disease states warrants striking levels of efforts in correlating between disease and metabolites, exploring the comprehensive metabolite databases when conducting metabolomics analyses, and detailed biological knowledge of potential receptor partners from the host for identified microbial metabolites. These scientific standards are unfortunately not often reached. Additionally, while some research groups have compiled databases of bacterial metabolites or biosynthetic gene clusters, these are far from commonplace or standardized.^61,62^ Thus, steps toward generation of more complete and standardized tools for metabolic analysis would represent a preliminary step toward generating more studies like those detailed in this review. Additionally, with database compilation and additional *in silico* research, networks of known bacterial metabolite/receptor interactions may be constructed to aid the further elucidation of microbial metabolites important to human health and disease.

Furthermore, interpretability of pilot studies such as these may limit their use in application. While these studies identify new and exciting microbial products that may have profound effect on host metabolism, the majority of these studies represent the primary characterization of the bacterial metabolite/host receptor relationship they are describing. This elevates the potential for results that may not be replicable under all conditions or effects that may be attributed to incorrect mechanistic explanations. For instance, microbially manufactured inosine was found to promote the efficacy of CD 8^+^ T cell-based immunotherapy by Mager et al., who attributed this effect to A_2A_R mediation.^19^ However, a study published at roughly the same time in Nature Metabolism found the effect of immunotherapy may be boosted by inosine via a different mechanism, namely that CD 8^+^ T cells may utilize it as an additional carbon source for energy production.^63^ This study found profound increases in immunotherapy effectiveness while inosine was added to tumor cells that were not able to utilize inosine as an energy substrate.^63^ Interestingly, they observed little effect of inosine to stimulate CD 8^+^ T cells when applied to MC-38 tumor cells, the same cells Mager et al. had success with.^63^ This illustrates the point that while identification of novel bacterial metabolites is a promising research direction, corroboration of observed effects of these metabolites is also crucial. In contrast to inosine and immunotherapy, subsequent studies concerning p-Cresol and its effect on autism promotion have served to fully corroborate the research conducted by Bermudez-Martin et al. Several subsequent studies have observed similar behavioral phenotypes or impaired neural processes in animal models.^64,65^ In cases where only one piece of primary literature is available detailing the effect of a metabolite, it is important to consider not only statistical significance but effect size and corroboration of the effect in multiple cell lines or organisms to promote confidence in study results. Additionally, some studies we surveyed include reproducibility statements or checklists that may further the ability to reproduce a study. For example, Liu et al. include a reproducibility statement which gives further transparency concerning study protocols.^15^ Meanwhile, Mager et al. include a Materials Design Analysis Reporting (MDAR) checklist, which is a standard, but rarely used, reporting tool that may greatly assist in study reproduction.^19,66^ Consistent use of this checklist, especially in pilot studies, may significantly improve study reproducibility.

While identifying novel receptor targets and disease states for which SCFAs and tryptophan metabolites elicit significant effects may certainly continue, further exploration of more novel metabolites such as those we have outlined likely will have more groundbreaking effects on the understanding of how the microbiota may influence human health. Furthermore, additional mechanistic analyses focused on these novel metabolites may confirm the effects postulated by the studies we have detailed. With this further verification, steps may be undertaken to translate these findings to clinical settings, where metabolites such as TMAO or inosine may be used as supplements to cancer immunotherapies, or those such as PAGln or p-Cresol may be investigated as therapeutic targets.

## Conclusions

Altogether, the breadth of microbial metabolites with modulatory effects on human metabolism or disease states is wider than previously imagined. Excitingly, these metabolites have been discovered to modulate inflammatory processes in the intestines, obesity, CVD, response to cancer immunotherapy, or even neurological processes. These metabolites may be quickly identified using high throughput genomics or metabolomics techniques, associated with particular bacterial species, and subsequently examined for receptor-mediated effects in known systems. It is also worth note that these metabolites can be significantly affected by dietary composition as well as taxonomic composition of the gut microbiome. Additionally, their impacts may be taken advantage of by the concomitant administration of drugs or therapies such as immunotherapy in the context of cancer treatment. While we have identified eight recent studies and seven metabolites to be of paramount interest, this list is by no means exhaustive or indicative of the count of total microbial metabolites that may impact host health. With further mechanistic studies following similar workflows, the library of bacterial metabolites with confirmed modulatory properties for human health and disease states will expand, allowing for more in-depth characterization of the gut microbe-host interactions.

## Data Availability

Data sharing is not applicable to this article as no new data were created or analyzed in this study.
